# Supplementary material for: Learning curve and surgical outcome of robotic assisted colorectal surgery with ERAS program
Source: Sci Rep. 2022 Nov 29;12:20566. doi: 10.1038/s41598-022-24665-w (PMC9709162; doi:10.1038/s41598-022-24665-w)
Supplement: Supplementary file 2 — Supplementary Information 2. [file 41598_2022_24665_MOESM2_ESM.zip › RAL Raw data-Ñ[▒K/081/hs_ñw╝╨░O▒Kñσ.pdf]

正式摘要(電子簽章：000000216659494)

W52 / W52 ☐英文標題(注意:無英文姓名-請補病人基本檔!) ☐【讀取】憑證

臺中榮民總醫院
出院病歷摘要

轉入號： 轉出號：

[redacted]

診斷

- 入院 :R-S colon cancer
- 出院 :R-S colon cancer, s/p Robot Assisted Colectomy on 2021/5/8

主訴

- bloody stool admitted for Robot Assisted Colectomy

病史

- The 39-year-old female suffered from bloody stool since last year. She had received hemorrhoid rubber band ligation and bloody stool still noted after ligation. She went to LMD for colonscope this March, which showed malignancy and she was transferred to our hospital. She denied bowel habbit change or dizziness. Weight loose about 2-3 kg was noted in 6 months. Therefore, after discussed with the doctor, she agreed to arrange Robot Assisted Colectomy and admitted for further care.

體檢發現

- Anal inspection:
  Recto digital examination:
  Sigmoidoscopy:
  Anoscopy:
  Abdomen:
  - General appearance :
    - easy looking , conscious clear , without cardiopulmonary distress
    - Vital signs自動帶入date 體溫(BT) 脈搏(P) 呼吸(R) 血壓(BP) 身高(BH) 體重(BW) BMI
      20210410 36.5℃ 93次/分 20次/分 134/60mmHg 147.6cm 48.2kg 22.1
    - Vital signs: B.P. 134/60 mmHg , P.R. 93 /min , R.R. 20 /min , B.T. 36.5 ℃
  - HEENT :
    - Eyes: no anemic conjunctiva, no icteric sclera, normal EOM
    - ENT: no otorrhea, no rhinorrhea, no erythematous mucosa, no injected tonsil, no deformity
  - Neck: supple, freely movable, trachea midline,no deformity, no engorged jugular vein , no palpable thyroid or lymph node enlargement
  - Chest:
    - Inspection: Grossly normal, symmetric and fully expansion, no accessory muscle used, no operation scar, no spider angiomata, no gynecomastia
    - Palpation: no palpable mass or nodule, no subcutaneous emphysema, no tenderness
    - Ausculation: clear breathing sound
  - Heart:
    - Palpation: no thrill, no heave
    - Percussion: normal heart size
    - Ausculation: regular heart beat, normal S1, S2, no gallop, no murmur
  - Extremities: No deformity, full ranged of motion without limitation, no clubbing finger or nail cyanosis, no pitting edema, no ulcer or pigmentation, no obvious skin lesion, no varicose vein, no bone, joint or muscle tenderness

  CRS CONDITION
  - Karnofsky performance status: (100) Normal, no complaints, no evidence of disease
  - body weight : 48 kg , height : 147 cm
  - CEA: 1849 ng/ml

手術日期及方法

- 手術日期及方法20210508 Robot Assisted Colectomy(L)

住院治療經過

- After admission, under no contraindication of surgery, Robot Assisted Colectomy was done smoothly on 2021/5/8. After surgery, no complication noted. Under stable condition, the patient was discharge and arranged OPD followed up.

合併症

一般檢查

- 累積報告：

DATE	TIME	ALB	TP	BIL.T	BIL.D	ALKP	AST	ALT	LDH	CHOL	TG	HDL-C	LDL	UA	NH3	AMY
1100508	1322	3.9		1.2	0.3	65	14	9								

DATE	TIME	WBC	RBC	HGB	HCT	MCV	PLT	BLST	NEUT	BAND	SEG	LYM	MON	EOS	BAS
1100508	1322	16950	4.36	12.1	37.5	86.0	285		96.6			2.7	0.6	0.0	0.1

DATE	TIME	NA	K	CL	CA	BUN	CREAT	Mg	P	CRP	ESR	PCT	LIPASE	LACTATE
1100508	1322	139	4.0	107	9.2	7	0.73							

DATE	TIME	GLUCOSE	PAB	TRF	ZN	CU	KETONE	CK	CKMB	PT-P	PT-C	APTT-P	APTT-C	TRO-T
1100508	1322	143												

特殊檢查

病理報告

其它

出院時情況

- 3.改門診治療

出院指示

出院計劃

主治醫師_2062F 蔣鋒帆_______________ / 住院醫師_9760F 趙英如_______________

正式摘要**(電子簽章：000000216659494)**

W52 / W52

doctorRank:B
